# Descriptive Analysis of Good Clinical Practice Inspection Findings from U.S. Food and Drug Administration and European Medicines Agency

**DOI:** 10.1007/s43441-022-00417-w

**Published:** 2022-05-24

**Authors:** Jenn W. Sellers, Camelia M. Mihaescu, Kassa Ayalew, Phillip D. Kronstein, Bei Yu, Yang-Min Ning, Miguel Rodriguez, LaKisha Williams, Ni A. Khin

**Affiliations:** 1 Division of Clinical Compliance Evaluation, Office of Scientific Investigations, Office of Compliance, Center for Drug Evaluation and Research, United States Food and Drug Administration, 10903 New Hampshire Ave, White Oak Building 51, Room 5324, Silver Spring, MD 20993 USA; 2 Inspections Office, Quality and Safety of Medicines Department, European Medicines Agency (EMA), Domenico Scarlattilaan 6, 1083 HS Amsterdam, The Netherlands

**Keywords:** Good clinical practice inspection, Inspection finding, Bioresearch monitoring, Clinical investigator, Sponsor

## Abstract

**Background:**

The United States Food and Drug Administration (FDA) and European Medicines Agency (EMA) have collaborated in good clinical practice (GCP) inspections since September 2009. The two agencies operate under different regulatory frameworks for GCP oversight. No systematic assessments of GCP inspection findings have been reported.

**Methods:**

We identified common inspections of clinical investigators, sponsors, and contract research organizations conducted by both agencies in support of marketing applications that had the same trial data submitted between 2009 and 2015. We grouped inspection findings into deficiency areas. We reviewed and compared these findings and calculated concordance rate for each deficiency area.

**Results:**

Twenty-six clinical investigator sites and 23 sponsors/contract research organizations were inspected by both agencies in support of 31 marketing applications during this period. For FDA, the most common GCP findings were deficiencies related to Protocol Compliance for clinical investigator inspections and Trial Management issues for sponsor/contract research organization inspections. For EMA, deficiencies related to Documentation (including Trial Master File) were the most common findings for both clinical investigator and sponsor/contract research organization inspections. There was high concordance, of approximately 90%, for deficiencies related to Protocol Compliance for clinical investigator inspections and Trial Management for sponsor/contract research organization inspections between the two agencies. There was a concordance rate of about 70% for Documentation deficiencies for both clinical investigator and sponsor/contract research organization GCP inspections.

**Conclusion:**

GCP inspection findings from 49 common clinical investigator and sponsor/contract research organization inspections were comparable, providing support for continued FDA-EMA GCP collaboration.

## Introduction

Good clinical practice (GCP) inspections are conducted by regulatory agencies to assess data integrity and to safeguard the rights, safety, and well-being of study participants as well as to ensure trials are conducted in compliance with GCP and applicable laws and regulations [1–6]. challenges associated with the globalization of clinical trials, FDA and EMA began a GCP collaboration in 2009 to conduct collaborative GCP inspections; conduct periodic information exchanges on GCP-related activities; and share information on interpretation of GCP. This collaboration allowed for a better understanding of each other’s inspection procedures [13]. Over time, this collaboration has expanded to include the regular exchange of inspection related information and the sharing of best inspection practices [14].

FDA and EMA operate under different regulatory frameworks for GCP inspections. For FDA’s Center for Drug Evaluation and Research, the assessment of GCP compliance and data integrity for marketing applications is performed by the Office of Scientific Investigations in collaboration with the Office of New Drugs and the Office of Regulatory Affairs. The GCP inspections are conducted by the FDA investigators under the agency wide bioresearch monitoring program using the 21 Code of Federal Regulations for clinical investigators and sponsors/contract research organizations. The basis for FDA inspection findings is 21 Code of Federal Regulations [1, 15]. These GCP inspections utilize a data-focused approach and verify individual subject level data and clinical trial conduct at investigator sites as well as assess sponsor/contract research organizations oversight responsibilities [1, 16]. For FDA, ICH E6 is guidance. In the European Union, in the context of the centralized procedure, GCP inspections are requested by the Committee for Medicinal Products for Human Use (CHMP), coordinated by EMA, and conducted by inspectors from the individual European Union member states following European Union laws, applicable national/local laws, and the International Council for Harmonization (ICH) guideline on good clinical practice (ICH E6) [2, 6]. The basis for the majority of EMA inspection findings is the ICH E6 guideline. EMA’s inspections cover GCP systems and processes in addition to data verification [6, 16].

In this paper, we report on a comparison of GCP findings from common sites inspected by both EMA and FDA covering the same trial data that was received in support of pre-approval applications. We also discuss the results and their implications.

## Methods

### Data Sources and Identification

The following data sources were used for this project: FDA and EMA internal databases, FDA’s establishment inspection reports and clinical inspection summaries, and EMA’s individual inspection reports and integrated inspection reports. The steps below were followed in order to identify GCP inspection findings:

1. Shared applications, defined as the same applications with the same study data submitted to both agencies for marketing authorization between January 1, 2009 and December 31, 2015, were identified.
2. Common inspections, defined as inspections conducted by both agencies at the same sites (clinical investigators, sponsors, or contract research organizations) for the same protocols for the shared applications, were then identified.
3. For these common inspections, FDA identified GCP findings by reviewing their establishment inspection reports and clinical inspection summaries. For EMA, GCP inspection findings were extracted from their internal inspection database, with quality audit checks using the individual inspection reports and integrated inspection reports.

All the GCP findings in the common inspections for the shared applications were collected for this study and grouped as described below.

### Grouping of GCP Findings

After FDA and EMA identified GCP findings for each of these common inspections, we grouped these findings by deficiency area using EMA’s list of GCP finding categories (Table 1) [17]. For the purposes of this paper, modifications were made to merge and better reflect the way the two agencies described inspection findings in their inspection reports. Briefly, the modifications consisted of:

1. Renaming several deficiency areas to make them more intuitive to all stakeholders by introducing the terms Protocol Compliance, Documentation, and Study Drug related findings as deficiency areas.
2. Combining Informed Consent, Independent Ethics Committee/Institutional Review Board, and Subject Protection into Human Subject Protection for the purpose of including all findings related to rights, safety and well-being of study participants under a single deficiency area.
3. Placing the findings unique to each regulatory agency (such as Form FDA 1572 [18] and financial disclosure [19] under 21 Code of Federal Regulations for FDA) under deficiency area of Regulatory Issues.

**Table 1:** Good clinical practice inspection findings by deficiency areas excluded from data analysis

Deficiency area	Deficiency sub-areas
Regulatory issues	Lack of local regulatory authority approval where the clinical site is located; approval/amendments/notifications to the regulatory authority; manufacturing/importing authorization; Form FDA 1572, Statement of the Investigator; FDA financial disclosure by Investigators
Laboratory/technical facilities	Certification and accreditation; validation; normal values/ranges/updates; shipment/storage/labeling/kit samples; accountability/traceability of samples; analysis/reporting (laboratory); technical validation
Computer system	Computer validation; audit trail and authorized access; physical security system and backup
Study drug	Manufacturing, packaging and labeling
Trial management	Protocol/case report form/diary/questionnaires design; Statistical analysis; Clinical study report
Subject protection	Insurance, indemnity and compensation to subjects; Payment to trial subjects; the design of the trial that could compromise subject protection

### Findings Excluded from Analysis

Prior to our analysis, a number of findings related to known operational and regulatory differences between the two agencies were excluded. For both clinical investigator and sponsor/contract research organization inspections, the findings related to the following deficiency areas (*subareas*) were excluded from analysis (Table 1):

1. Regulatory issues: this is specific to each agency such as Form FDA 1572, the Statement of Investigator Form [18] and financial disclosure requirements [19].
2. Laboratory/technical facilities: this is generally covered under separate programs for FDA; for example, assay validation, and sample storage.
3. Computer system: FDA was not covering computer system validation in sponsor/contract research organizations inspections during the study period of 2009–2015 [20, 21].
4. Study drug (*Manufacturing/Packaging/Labeling*): FDA’s GCP inspections do not cover the subareas of manufacturing and product packaging, which are generally covered under Good Manufacturing Practice inspections [6, 22]. Also, the regulatory requirements for labeling are different between the two agencies [6, 18, 23].
5. Trial management (*Study Protocol Design, Statistical Analysis and Clinical Study Report)*: FDA’s review process is different with regards to these subareas. FDA’s multidisciplinary review teams (including biostatisticians) are responsible for evaluating these subareas.
6. Human subject protection (Liability Insurance, Subject Compensation for Trial Related Injuries and The Design of the Trial that Could Compromise Subject Protection): These subareas were excluded as FDA inspections do not cover them.

In addition, the following findings were excluded because clinical investigator and sponsor/contract research organization inspections are inspected under different FDA compliance programs (Table 2) [24, 25].

1. For the clinical investigator inspection analysis, the findings under Trial Management were excluded because, according to FDA’s regulations, trial management is the responsibility of sponsor or entities to whom the sponsor has transferred regulatory obligations such as a contract research organization [25, 26].
2. For the sponsor/contract research organization inspection analysis, the findings under Human Subject Protection were excluded because the findings related to Human Subject Protection are cited under the clinical investigator who was responsible for the study [27].

**Table 2:** Good clinical practice inspection findings by deficiency areas included in data analysis

Deficiency area	Deficiency sub-areas
Protocol compliance#	Eligibility criteria
	Assessment of efficacy
	Safety reporting
	Reporting in case report form/diary as specified in the protocol
	Other protocol non-compliance not listed above
Trial management*	Data management
	Monitoring
	Document control
	Audit
Documentation#	Essential documents
	Source documentation
	Qualification and training
	Standard operating procedures
	Organization and personnel
	Facilities and equipment
	Randomization, blinding and codes of study drug
	Direct access to data
	Contracts and agreements
Study drug#	Drug accountability
	Supplying, storage, retrieving and destruction
	Prescription, administration and compliance
Human subject protection#,*	Informed consent • Presence of informed consent in the site • Informed consent process • Informed consent form content
	Independent ethics committee/institutional review board • Favorable opinion in the site • Opinion, amendments and notifications to the Independent Ethics Committee/Institutional Review Board • Composition, functions and operation
	Subject protection • Personal data protection • Safeguard of the safety and well-being of subject

*The findings in Trial Management were only included in the sponsor/contract research organization inspection analysis because it is the responsibility of sponsor or contract research organization. The findings in Human Subject Protection were only included in the clinical investigator inspection analysis because it is the responsibility of clinical investigator, according to the FDA regulations

^#^Name modifications from EMA good clinical practice finding categories:

“Protocol Compliance” = “Investigational Site”

“Documentation” = “General”

“Study Drug” = “Investigational Medicinal Products”

“Human Subject Protection” = “Informed Consent” + “Independent Ethics Committee/Institutional Review Board” + “Subject Protection”

### Concordance Analysis

After the analysis datasets were created, we reviewed and compared the GCP findings as well as calculated concordance rate for each deficiency area. We defined concordance as both agencies having identified one or more findings in the same deficiency area for a particular site. We calculated concordance rate by site and deficiency areas using the formula below:

$$\frac{Number\,of\,sites\,with\,concordance\,(had\,findings\,by\,both\,agencies)}{{Number\,of\,sites\,\,that\,had\,one\,or\,more\,findings}} \times 100\%$$

Non-concordance was defined as only one agency having findings in a certain deficiency area for a particular site. Due to the number of findings at the non-concordant sites, representative examples are provided in the Results.

## Results

### GCP Inspection Findings

**Fig. 1:**
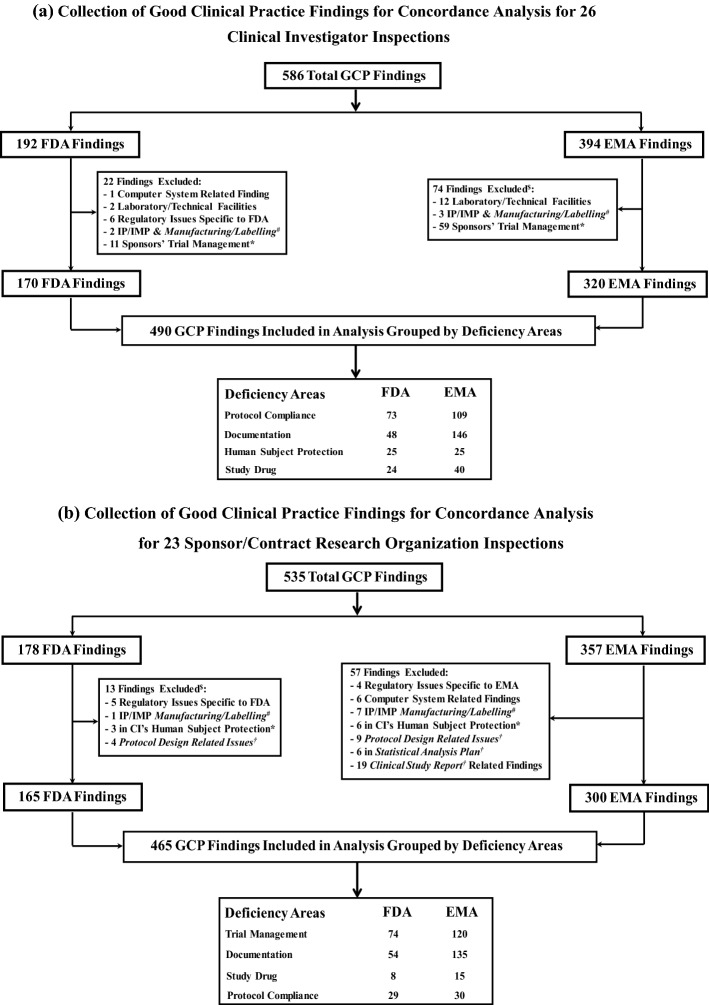
**a** Collection of Good Clinical Practice Findings for Concordance Analysis for 26 Clinical Investigator Inspections. *IP* Investigational Product, *IMP* Investigational Medicinal Product. There were no findings in the subareas of Subject Protection: Personal Data Protection,Insurance/Indemnity/Compensation to Subjects, and Payment to trial Subjects by either FDA or EMA # Subcategory of Study Drug (only IP/IMP manufacturing and labeling related findings were excluded); all other IP/IMP related findings are captured under Study Drug. *Excluded Sponsor’s responsibility of Trial Management findings cited in clinicals investigator inspection reports $EMA did not have findings in Computer System, or Regulatory Issues. **b** Collection of Good Clinical Practice Findings for Concordance Analysis for 23 Sponsor/Contract Research Organization Inspections. # Only IP/IMP manufacturing and labeling related findings were excluded (Subcategory of Study Drug); all other IP/IMP related findings are captured under Study Drug. *Findings related to Human Subject Protection were cited under clinical investigator inspections. †Protocol Design Related Issues including CRF, eDiary or Questionnaire designs (Subcategory of Trial Management); all other trial management related findings are captured under Trial Management by Sponsors and Contract Research Organizations. $ FDA did not have findings in Computer System, Statistical Analysis Plan, and Clinical Study Report. Figure 1 shows how the final data sets for concordance analysis are derived for 26 Clinical Investigator Inspections (Fig. 1a) and 23 sponsor/contract research organization inspections (Fig. 1b) by excluding known regulatory differences between the two agencies described in “Methods”

A total of 49 common GCP inspections were conducted by EMA and FDA in support of 31 shared marketing applications from 2009 through 2015. Twenty-six of the common GCP inspections were for inspections of clinical investigators and 23 were for sponsors/contract research organizations. For the 26 clinical investigator inspections, a total of 170 and 320 findings were included in the final dataset for FDA and EMA, respectively (Fig. 1a). For the 23 sponsor/contract research organization inspections, a total of 165 and 300 findings were included in the final dataset for FDA and EMA, respectively (Fig. 1b). An analysis of the difference in the number of inspection findings between the two agencies is beyond the scope of this study.

**Fig. 2:**
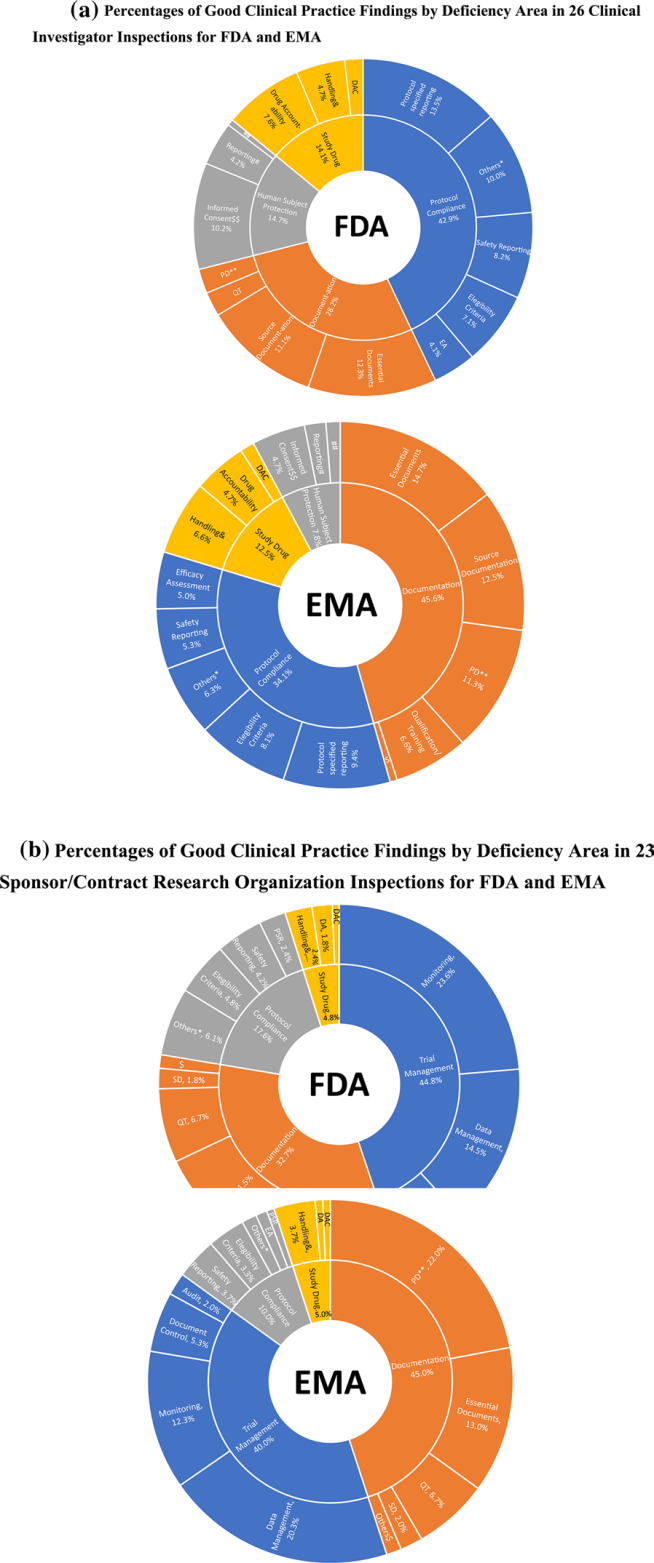
**a** Percentages of good clinical practice findings by deficiency area in 26 clinical investigator inspections for FDA and EMA. **b** Percentages of good clinical practice findings by deficiency area in 23 sponsor/contract research organization inspections for FDA and EMA. *DA* Drug accountability, *DAC* Drug administration and compliance, *EA* Efficacy assessment; *PD* Process documentation, *PSR* Protocol specified reporting, *QT* Qualification/training, *SD* Source documentation. *PC related findings not listed under other sub-areas. **including Organization and Personnel, Facilities and Equipment, SOPs, and Contracts/Agreement. $ Others: Documentation related findings not listed under other sub-areas. $$ including lack of informed consent in the site, informed consent process, and informed consent form. # IRB/IEC reporting: including lack of IEC/IRB favorable opinion in the site, Opinion/Amendments/Notifications to the IEC/IRB, and Composition, functions and operation. ## Others: Safeguard of the Safety and well-being of Subject & Supplying/storage/retrieving/destruction

For the clinical investigator inspections, for FDA, the most inspection findings were in the deficiency areas of Protocol Compliance (43%) and Documentation (28%); while for EMA, the most common findings were in the deficiency areas of Documentation (46%) and Protocol Compliance (34%) (Fig. 2a).

For sponsor/contract research organization inspections, for FDA, the most inspection findings were in the deficiency areas of Trial Management (45%) and Documentation (33%); while for EMA, the most common findings were in the deficiency areas of Documentation (45%) and Trial Management (40%) (Fig. 2b).

### Concordance for Clinical Investigator Inspections

**Fig. 3:**
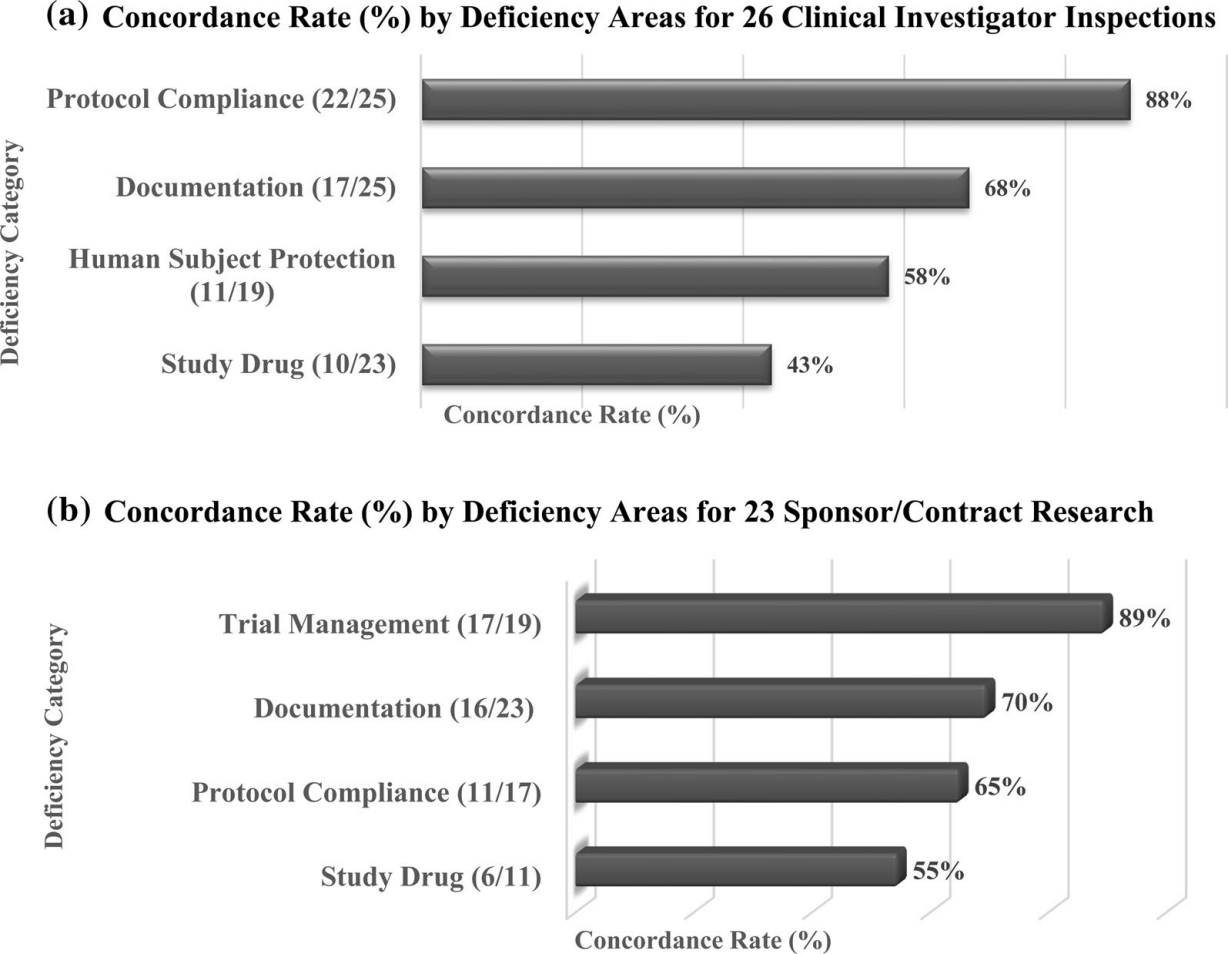
**a** Concordance rate (%) by deficiency areas for 26 clinical investigator inspections. **b** Concordance rate (%) by deficiency areas for 23 sponsor/contract research organization inspections. The concordance rate is calculated by the number of sites with concordance (had findings by both agencies) divided by the number of sites which had one or more findings in the same deficiency area times 100%

The concordance rates between the two agencies by site and deficiency area were calculated for each of the inspected sites (Fig. 3). Out of the 26 common clinical investigators inspected, 25 clinical investigator inspections had findings under the deficiency areas of Protocol Compliance and Documentation. Both agencies identified deficiencies related to Protocol Compliance at 22 of the 25 clinical investigator sites, a concordance rate of 88%. For the three non-concordant sites (one had findings by FDA and two had findings by EMA), 13 findings were identified, examples of which included FDA’s finding that a concomitant medication for one subject was not reported to the sponsor and EMA’s finding that an enrolled subject did not meet study eligibility criteria.

For Documentation deficiency area, both agencies identified findings at 17 of the 25 clinical investigator sites, making for concordance rate of 68%. All eight non-concordant sites had Documentation findings by EMA. The differing findings were mainly due to the following reasons: (1) FDA generally does not include sponsor responsibility related findings in its clinical investigator inspection reports [24] and (2) some findings reported by EMA were related to ICH-E6(R1) GCP requirements, [6] for which the FDA does not have parallel requirements under 21 Code of Federal Regulations. (1) (Table 3) Examples include delayed placement of qualification or training documentation in trial master file, and lack of adequate version control for essential documents [6].

For Human Subject Protection, 19 of the 26 sites had findings, with both agencies identifying findings at 11 of the 19 sites (concordance rate of 58%). Five of 8 non-concordant sites had findings by FDA and three had findings by EMA. One example of FDA’s findings was related to inadequate informed consent processes for two subjects [27]. An example of EMA’s findings at the three remaining non-concordant sites was sending a protocol amendment late to the Independent Ethics Committee [6].

Twenty-three [23] sites had Study Drug findings, with both agencies identifying findings at 10 of the 23 sites (concordance rate of 43%). Nine of 13 non-concordant sites had findings by EMA. An example of EMA findings included inadequate documentation of study drug shipment and late acknowledgement of receipt of study drug [6]. An example of FDA’s findings at the remaining 4 non-concordant sites included inadequate documentation of the amount of the study drug taken by one subject (see Table 3).

**Table 3:** Good clinical practice inspection regulatory references

Deficiency areas category/sub-categories	ICH E6 (R1) Section/CPMP/ICH/ 135/95	FDA code of federal regulations, Title 21 Part 312, 50, 56
*Trial management*		
Data management	Specifically requires that the data changes are documented and that there is no deletion of entered data (i.e., maintain an audit trail, data trail, edit trail) (5.5)	Has general language that a sponsor is required to select qualified clinical investigators (312.50), who are required to prepare and maintain adequate and accurate case histories (312.62)
Monitoring	Has specific requirements concerning the purpose of monitoring; the selection and qualifications of monitors; extent and nature of monitoring Monitor’s responsibilities; monitoring procedures, monitoring report and monitoring plan (5.18) The sponsor may consider establishing an independent data monitoring committee, to assess the progress of a clinical trial, including the safety data and the critical efficacy endpoints at intervals, and to recommend to the sponsor whether to continue, modify, or stop a trial. The independent data monitoring committee should have written operating procedures and maintain written records of all its meetings (5.5.2)	Has general language that the sponsor shall select a monitor qualified by training and experience to monitor the progress of the investigation (312.53); and is responsible for ensuring proper monitoring of the investigation(s) (312.56)
		No requirements for the use of data monitoring committee in trials except research studies in emergency settings in which the informed consent requirement is excepted (50.24)
Document control	Specific requirements of document identification and version history [8]	No specific requirement of version history
Audit	Gives detailed guidance on how sponsors should conduct site quality assurance audits	No specific requirements. However, many sponsors obtain independent audits/data verifications to determine the compliance with clinical trial sops and FDA regulations and verify the accuracy of the case reports
*Documentation*		
Essential documents	Trial Master File includes documents, for examples: • Insurance Statement • Subject Screening Log • Signature Sheet (to document signatures/initials of persons authorized to make CRF entries and corrections) • Pretrial and trial initiation monitoring report from sites • Subject screening and enrollment log/subject identification code list ICH specifically requires filing essential documents in a timely manner [8]	no specifics as Trial Master File but has different requirements of essential documents
Qualification and training	Requires documentation of GCP training The investigator(s) should be qualified by education, training, and experience to assume responsibility for the proper conduct of the trial, should meet all the qualifications specified by the applicable regulatory requirement(s), and should provide evidence of such qualifications through up-to-date curriculum vitae and/or other relevant documentation requested by the sponsor, the IRB/IEC, and/or the regulatory authority(ies) The investigator should be aware of, and should comply with, GCP and the applicable regulatory requirements (4.1)	Has similar requirements but does not require GCP training (312.53)
Standard operating procedures	The sponsor is responsible for implementing and maintaining quality assurance and quality control systems with written standard operating procedures Requires Monitoring procedures (Monitor Plan) and auditing procedures (5.18)	No specific requirements of standard operating procedures
Organization and personnel	Requires maintaining a delegation log. The investigator should maintain a list of appropriately qualified persons to whom the investigator has delegated significant trial-related duties (4.1.5)	The investigator is responsible for supervising any individual or party to whom the investigator delegates trial-related duties and functions conducted at the trial site; but documentation only requires a list of the names of the sub-investigators (312.53)
Contracts and agreements	ICH has specific requirement for signatures of documents (8.2.6)	Similar requirements in writing for transferred obligations but no specific mentioning of signatures (312.52)
Study drug		
Supplying/storage/retrieving/destruction	Investigational products should be manufactured, handled, and stored in accordance with applicable good manufacturing practice	A sponsor shall maintain adequate records showing the receipt, shipment, or other disposition of the investigational drug (312.57)
	Sponsor should determine for investigational medicinal product acceptable storage, temperatures, conditions during shipment The sponsor should have *written procedures* including adequate, safe receipt and handling	
	The sponsor should ensure that written procedures include instructions that the investigator/institution should follow for the handling and storage of investigational product(s) for the trial and documentation thereof. The procedures should address adequate and safe receipt, handling, storage, dispensing, retrieval of unused product from subjects, and return of unused investigational product(s) to the sponsor (or alternative disposition if authorized by the sponsor and in compliance with the applicable regulatory requirement(s)) (5.14)	
*Informed consent*		
Informed consent process/informed consent form	The written informed consent form should be signed and personally dated by the subject	Informed consent shall be documented by the use of a written consent form approved by the IRB and signed and dated by the subject or the subject's legally authorized representative at the time of consent (50.27) Allows to use the short form written consent document to be signed by the subject or the representative; a witness to the oral presentation sign both the short form and a copy of the summary; the person who conducted the informed consent discussion sign a copy of the summary (50.27)
	Or by the subject’s legally acceptable representative, and by the person who conducted the informed consent discussion prior to a subject’s participation in the trial (4.8.8., 4.8.9)	
	Requires that the subject receive a signed and dated copy of the written informed consent (4.8.11)	Requires that a copy be given to the subject but does not state that it must be a signed copy (50.27)
	Detailed specific explanations are required to give to study subjects during the informed consent discussion such as: Trial treatments and probability of random assignment The anticipated prorated payment, if any, to the subject for participating in the trial	Requires notifying subject that clinical trial information has been or will be submitted for inclusion in the clinical trial registry and need to contain the Clinical Trial registry language (50.25)
	access of medical records by monitor(s), the auditor(s), the IRB/IEC, and the regulatory authority(ies) to the subject's original medical records (4.8)	Requires stating the possibility that the Food and Drug Administration may inspect the records (50.25)
*Institutional review boards/independent ethics committees*		
Opinion/amendments/notifications to the Institutional Review Boards/Independent Ethics Committees	The investigator should not implement any deviation from, or changes of the protocol without agreement by the sponsor and prior review and documented approval/favorable opinion from the Institutional Review Boards/Independent Ethics Committees of an amendment, except where necessary to eliminate an immediate hazard(s) to trial subjects, or when the change(s) involves only logistical or administrative aspects of the trial (e.g., change in monitor(s), change of telephone number(s)) (3, 4.5)	The investigator shall also assure that he or she will promptly report to the Institutional Review Board all changes in the research activity and all unanticipated problems involving risk to human subjects or others, and that he or she will not make any changes in the research without Institutional Review Board approval, except where necessary to eliminate apparent immediate hazards to human subjects. (56.109, 312.66)

Table 3 shows the good clinical practice inspection regulatory differences of ICH E6 (R1) and FDA Code of Federal Regulations, Title 21 Part 312, 50, 56 for the study period of 2009–2015. CPMP Committee for Proprietary Medicinal Products, which was replaced by the current name Committee for Medicinal Products for Human Use (CHMP) in May 2004

### Concordance for Sponsor/Contract Research Organization Inspections

The concordance rates between the two agencies by deficiency area for the common sponsor/contract research organization are provided in Fig. 3b.

Out of the 23 common sponsor/contract research organizations inspected, 19 had findings related to Trial Management. Both agencies identified deficiencies related to Trial Management at 17 of these 19 (concordance rate of 89%). The two non-concordant sites had Trial Management findings reported by EMA. These differing findings were mostly due to EMA requirements linked to ICH-E6 with regard to timeliness of maintenance of essential documents and a delay in establishing the monitoring plan [6] (Table 3).

For the Documentation deficiency area, all 23 inspections had findings. Both agencies identified findings at 16 of the 23 sites (concordance rate of 70%). The seven non-concordant sites had findings by EMA. Example findings include deficiencies in completeness of documentation in trial master file, and lack of updating standard operating procedures in a timely manner.

For Protocol Compliance, of 17 sites with findings, 11 were identified by both agencies (concordance rate of 65%). Five of 6 non-concordant sites had findings by EMA, and one had findings by FDA. Examples of EMA findings included an inadequate process to collect and review protocol deviations. FDA’s finding at the remaining non-concordant site was that radiographs were taken out of the scheduled visit windows.

Eleven sites had findings related to Study Drug, and both agencies identified findings at 6 of these 11 sites (concordance rate of 55%). Four of 5 non-concordant sites had findings by EMA, and one had findings by FDA. An example of EMA findings included inadequate management and oversight of study drug shipment to clinical investigator sites. FDA’s finding at the remaining non-concordant site was that study drugs were diluted before administration.

## Discussion

In this paper, we described similarities and differences in findings for common inspections between EMA and the FDA over a 6-year period of GCP collaboration. For deficiencies related to Protocol Compliance for common clinical investigator inspections and Trial Management for common sponsor/contract research organizations inspections, there was high concordance of ~ 90%. There was a concordance rate of ~ 70% for Documentation deficiencies for both clinical investigator and sponsor/contract research organizations inspections. The concordance rate of 70% in Documentation is encouraging given the known differences in the operation and regulatory requirements between the two agencies.  The discordance in Documentation deficiency area for clinical investigators and sponsors/contract research organizations inspections was in large part due to differences primarily related to trial master file and signature requirements on a number of essential documents like contracts and standard operating procedures by EMA (Table 3).

There were various limitations to our study. This was a retrospective analysis of GCP inspection data. It is important to note that the trial records reviewed/audited at any inspected site could vary between the two agencies. In some cases, even if the same trial participant records were reviewed, it was possible that not all records were completely examined by both agencies. The inspections might not have covered exactly the same study records (for example, source records, administrative records) by the two agencies. There were other factors that could have affected the differences in inspection findings, such as the number of inspectors who participated in each inspection, the number of hours spent by each inspector, the training, background, and expertise of GCP inspectors. Due to the limitations described above, the definition of concordance appears meaningful in comparing the deficiencies in GCP inspections between the two agencies.

## Conclusion

GCP inspection findings from 49 common clinical investigator and sponsor/contract research organization inspections were comparable. The analysis provides support for our existing practice of sharing information between the two agencies for GCP inspection planning purposes as well as for the exchange of inspection reports. Also, this allows for the broadening of inspection coverage and avoiding duplicate inspections. This in turn permits more efficient utilization of the finite resources available for GCP inspections.

Recently, EMA-FDA GCP collaboration has been expanded to include Pharmaceuticals and Medical Devices Agency (PMDA) Japan as trilateral GCP collaboration [28]. Moving forward, EMA-FDA-PMDA plan to enhance their existing GCP collaboration in terms of continuous process improvement through guidance development and joint training programs, strengthen regulatory convergence, and form global GCP inspection collaboration in support of shared marketing application review. Joint GCP workshops, global regulatory engagement at professional society conferences, scientific exchange programs and ongoing participation in the ICH-E6(R3) GCP renovation effort would be beneficial in achieving these goals [29–32].

## References

[1] US Title 21 Code of Federal Regulations (CFR). Available from: https://www.accessdata.fda.gov/scripts/cdrh/cfdocs/cfcfr/CFRSearch.cfm. Accessed 1 Apr 2022.

[2] Directive 2001/20/EC OF the European Parliament and of the Council of 4 April 2001 on the approximation of the laws, regulations and administrative provisions of the member states relating to the implementation of good clinical practice in the conduct of clinical trials on medicinal products for human use. Available from: https://www.eumonitor.eu/9353000/1/j9vvik7m1c3gyxp/vitgbgi8z5z8. Accessed 1 Apr 2022. 16276663

[3] Commission Directive 2005/28/EC of 8 April 2005 laying down principles and detailed guidelines for good clinical practice as regards investigational medicinal products for human use, as well as the requirements for authorization of the manufacturing or importation of such products. Available from: https://eurlex.europa.eu/LexUriServ/LexUriServ.do?uri=OJ:L:2005:091:0013:0019:en:PDF. Accessed 1 Apr 2022.

[4] Regulation No 536/2014 of the European Parliament and of the Council of 16 April 2014 on clinical trials on medicinal products for human use, and repealing Directive 2001/20/EC. Available from: https://eur-lex.europa.eu/legal-content/EN/TXT/PDF/?uri=CELEX:32017R0556&rid=1. Accessed 1 Apr 2022.

[5] Commission Implementing Regulation (EU) 2017/556 of 24 March 2017 on detailed arrangements for the good clinical practice inspection procedures pursuant to Regulation (EU) No 536/2014 of the European Parliament and of the Council. Available from: https://eur-lex.europa.eu/legal-content/EN/TXT/PDF/?uri=CELEX:32017R0556&rid=1. Accessed 1 Apr 2022.

[6] ICH Topic E6 (R1). Guideline for Good Clinical Practice. Available from: https://www.ema.europa.eu/en/documents/scientific-guideline/ich-e6-r1-guideline-good-clinical-practice_en.pdf. Accessed 1 Apr 2022.

[7] Thiers FA, Sinskey AJ, Berndt ER (2008). Trends in the globalization of clinical trials. Nat Rev Drug Discov.

[8] Glickman SW, McHutchison JG, Peterson ED, Cairns CB, Harrington RA, Califf RM (2009). Ethical and scientific implications of the globalization of clinical research. N Engl J Med.

[9] George M, Selvarajan S, Suresh-Kumar S, Dkhar SA, Chandrasekaran A (2013). Globalization of clinical trials—where are we heading?. Curr Clin Pharmacol.

[10] Richter TA (2014). Clinical research: a globalized network. PLoS ONE.

[11] Shenoy P (2016). Multi-regional clinical trials and global drug development. Perspect Clin Res.

[12] Drain PK, Parker RA, Robine M, Holmes KK, Bassett IV (2018). Global migration of clinical research during the era of trial registration. PLoS ONE.

[13] EMEA-FDA GCP initiative. Available from: https://www.ema.europa.eu/en/human-regulatory/research-development/compliance/good-clinical-practice. Accessed 1 Apr 2022.

[14] EMA partners-networks. Available from: https://www.ema.europa.eu/en/partners-networks/international-activities/bilateral-interactions-non-eu-regulators/united-states. Accessed 1 Apr 2022.

[15] US Title 21 Code of Federal Regulations (CFR), Part 56 (Institutional Review Boards). Available from: https://www.accessdata.fda.gov/scripts/cdrh/cfdocs/cfcfr/CFRSearch.cfm?CFRPart=56&showFR=1. Accessed 1 Apr 2022.

[16] Khin NA, Francis G, Mulinde J, Grandinetti C, Skeete R, Yu B (2020). Data integrity in global clinical trials: discussions from joint US food and drug administration and UK medicines and healthcare products regulatory agency good clinical practice workshop. Clin Pharmacol Ther.

[17] EMA. Classification and analysis of the GCP inspection findings of GCP inspections conducted at the request of the CHMP (Inspection reports to EMA 2000–2012). Available from: https://www.ema.europa.eu/en/documents/other/classification-analysis-good-clinical-practice-gcp-inspection-findings-gcp-inspections-conducted_en.pdf. Accessed 1 Apr 2022.

[18] US Title 21 Code of Federal Regulations (CFR), Part 312 (INVESTIGATIONAL NEW DRUG APPLICATION). Available from: https://www.accessdata.fda.gov/scripts/cdrh/cfdocs/cfcfr/CFRSearch.cfm?fr=312.53. Accessed 1 Apr 2022.

[19] US Title 21 Code of Federal Regulations (CFR), Part 54 (financial disclosure by clinical investigators). Available from: https://www.accessdata.fda.gov/scripts/cdrh/cfdocs/cfcfr/CFRsearch.cfm?CFRPart=54. Accessed 1 Apr 2022.

[20] US Title 21 Code of Federal Regulations (CFR), Part 11 (electronic records; electronic signatures). Available from: https://www.accessdata.fda.gov/scripts/cdrh/cfdocs/cfcfr/CFRSearch.cfm?CFRPart=11. Accessed 1 Apr 2022.

[21] FDA. Use of electronic records and electronic signatures in clinical investigations under 21 CFR Part 11—Questions and Answers. Guidance for Industry, JUNE 2017. Available from: https://www.fda.gov/regulatory-information/search-fda-guidance-documents/use-electronic-records-and-electronic-signatures-clinical-investigations-under-21-cfr-part-11. Accessed 1 Apr 2022.

[22] EU. The rules governing medicinal products in the European Union. Volume 4. Good Manufacturing Practice. Available from: https://ec.europa.eu/health/sites/health/files/files/eudralex/vol-4/2017_11_22_guidelines_gmp_for_atmps.pdf. Accessed 1 Apr 2022.

[23] ICH 5.13.1, Sponsor: Manufacturing, Packaging, Labelling, and Coding Investigational Product(s). Available from: https://ichgcp.net/5-sponsor. Accessed 1 Apr 2022.

[24] FDA. Compliance Program Guidance Manual (CPGM), Clinical Investigators and Sponsor-Investigators. Available from: https://www.fda.gov/media/75927/download. Accessed 1 Apr 2022.

[25] FDA. Compliance Program Guidance Manual (CPGM) sponsor. Available from: https://www.fda.gov/media/75916/download. Accessed 1 Apr 2022.

[26] 21CFR, Part 312.52, transfer of obligation to CRO. Available from: https://www.accessdata.fda.gov/scripts/cdrh/cfdocs/cfcfr/cfrsearch.cfm?fr=312.52. Accessed 1 Apr 2022.

[27] US Title 21 Code of Federal Regulations (CFR), Part 50 (requirements of informed consent). Available from: https://www.accessdata.fda.gov/scripts/cdrh/cfdocs/cfcfr/CFRSearch.cfm?CFRPart=50. Accessed 1 Apr 2022.

[28] EMA-FDA-PMDA Trilateral GCP Feasibility Pilot Report. Available from: https://www.fda.gov/media/145550/download. Accessed 1 Apr 2022.

[29] FDA. Real-time oncology review (RTOR) pilot program. Available from: https://www.fda.gov/about-fda/oncology-center-excellence/real-time-oncology-review. Accessed 1 Apr 2022.

[30] Pharmaceutical Inspection Convention, Pharmaceutical Inspection Co-operation Scheme (PIC/S) Inspectorates Academy (PIA). Working Group on Good Clinical and Pharmacovigilance Practices. Available from: https://picscheme.org/en/pia-pic-s-training-expert-circles-training-working-group-on. Accessed 1 Apr 2022.

[31] ICH E6 (R2). Available from: https://www.ema.europa.eu/en/documents/scientific-guideline/ich-e-6-r2-guideline-good-clinical-practice-step-5_en.pdf. Accessed 1 Apr 2022.

[32] ICH Reflection Paper on GCP Renovation. Efficacy Guidelines E6(R3) EWG endorsed documents. Available from: https://www.ich.org/page/efficacy-guidelines. Accessed 1 Apr 2022.

