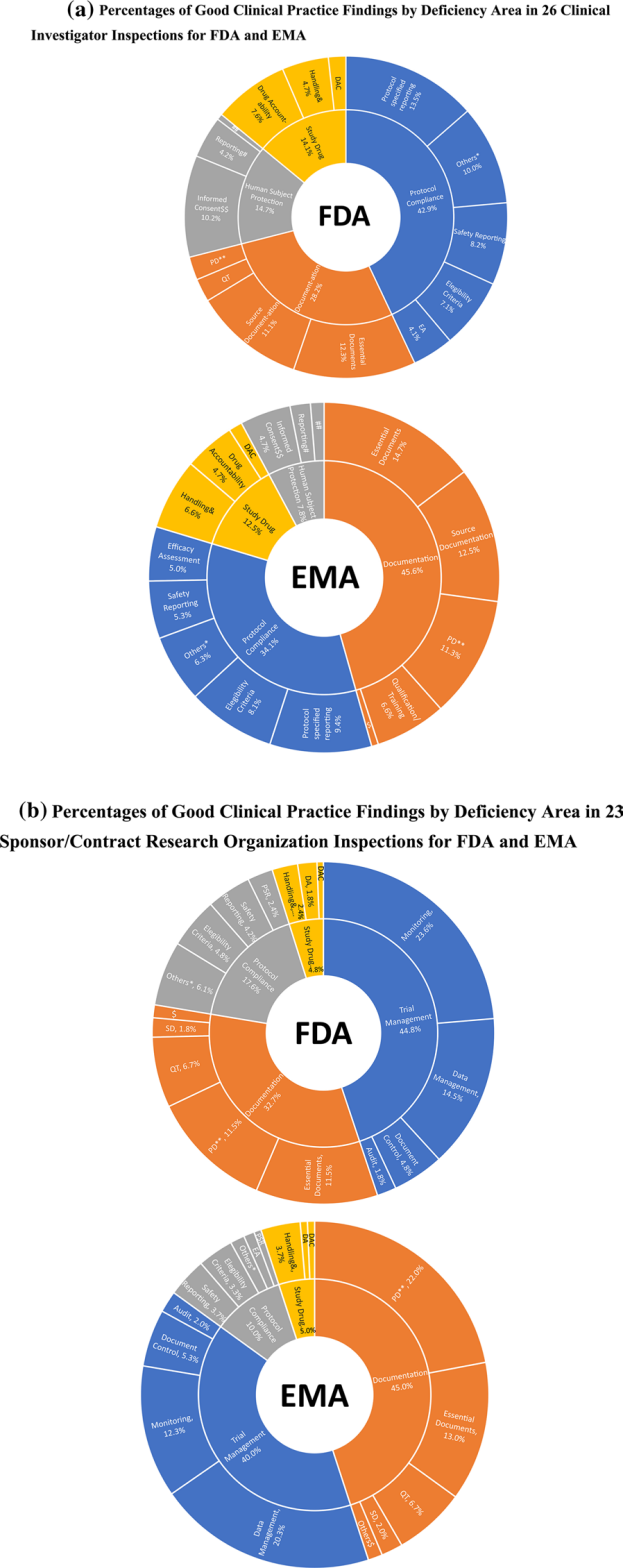# Publisher Correction: Descriptive Analysis of Good Clinical Practice Inspection Findings from U.S. Food and Drug Administration and European Medicines Agency

**DOI:** 10.1007/s43441-022-00423-y

**Published:** 2022-05-30

**Authors:** Jenn W. Sellers, Camelia M. Mihaescu, Kassa Ayalew, Phillip D. Kronstein, Bei Yu, Yang-Min Ning, Miguel Rodriguez, LaKisha Williams, Ni A. Khin

**Affiliations:** 1 Division of Clinical Compliance Evaluation, Office of Scientific Investigations, Office of Compliance, Center for Drug Evaluation and Research, United States Food and Drug Administration, 10903 New Hampshire Ave, White Oak Building 51, Room 5324, Silver Spring, MD 20993 USA; 2 Inspections Office, Quality and Safety of Medicines Department, European Medicines Agency (EMA), Domenico Scarlattilaan 6, 1083 HS Amsterdam, The Netherlands

## Correction to: Therapeutic Innovation & Regulatory Science 10.1007/s43441-022-00417-w

Due to a processing error a portion of Fig. 2b is missing in the original article. The corrected figure follows: