# Correction to: Descriptive Analysis of Good Clinical Practice Inspection Findings from U.S. Food and Drug Administration and European Medicines Agency

**DOI:** 10.1007/s43441-022-00427-8

**Published:** 2022-06-06

**Authors:** Jenn W. Sellers, Camelia M. Mihaescu, Kassa Ayalew, Phillip D. Kronstein, Bei Yu, Yang-Min Ning, Miguel Rodriguez, LaKisha Williams, Ni A. Khin

**Affiliations:** 1 Division of Clinical Compliance Evaluation, Office of Scientific Investigations, Office of Compliance, Center for Drug Evaluation and Research, United States Food and Drug Administration, 10903 New Hampshire Ave, White Oak Building 51, Room 5324, Silver Spring, MD 20993 USA; 2 Inspections Office, Quality and Safety of Medicines Department, European Medicines Agency (EMA), Domenico Scarlattilaan 6, 1083 HS Amsterdam, The Netherlands

## Correction to: Therapeutic Innovation & Regulatory Science 10.1007/s43441-022-00417-w

In the original article a phrase was omitted from the first paragraph of the Introduction. The corrected paragraph follows:

Good clinical practice (GCP) inspections are conducted by regulatory agencies to assess data integrity and to safeguard the rights, safety, and well-being of study participants as well as to ensure trials are conducted in compliance with GCP and applicable laws and regulations [1–6]. Clinical trials have become increasingly global in the past two decades [7–12]. To address challenges associated with the globalization of clinical trials, FDA and EMA began a GCP collaboration in 2009 to conduct collaborative GCP inspections; conduct periodic information exchanges on GCP-related activities; and share information on interpretation of GCP. This collaboration allowed for a better understanding of each other’s inspection procedures [13]. Over time, this collaboration has expanded to include the regular exchange of inspection related information and the sharing of best inspection practices [14].

